# Imaging practice in low-grade gliomas among European specialized centers and proposal for a minimum core of imaging

**DOI:** 10.1007/s11060-018-2916-3

**Published:** 2018-07-10

**Authors:** Christian F. Freyschlag, Sandro M. Krieg, Johannes Kerschbaumer, Daniel Pinggera, Marie-Therese Forster, Dominik Cordier, Marco Rossi, Gabriele Miceli, Alexandre Roux, Andrés Reyes, Silvio Sarubbo, Anja Smits, Joanna Sierpowska, Pierre A. Robe, Geert-Jan Rutten, Thomas Santarius, Tomasz Matys, Marc Zanello, Fabien Almairac, Lydiane Mondot, Asgeir S. Jakola, Maria Zetterling, Adrià Rofes, Gord von Campe, Remy Guillevin, Daniele Bagatto, Vincent Lubrano, Marion Rapp, John Goodden, Philip C. De Witt Hamer, Johan Pallud, Lorenzo Bello, Claudius Thomé, Hugues Duffau, Emmanuel Mandonnet

**Affiliations:** 1 Department of Neurosurgery, Medical University of Innsbruck, Anichstrasse 35, 6020 Innsbruck, Austria; 2 Department of Neurosurgery, Klinikum rechts der Isar, Technische Universität München, Munich, Germany; 3 Department of Neurosurgery, Goethe University Hospital, Frankfurt am Main, Germany; 4 Department of Neurosurgery, Universitätsspital Basel, Basel, Switzerland; 5 Neurosurgical Oncology Unit, Humanitas Research Hospital, IRCCS, Milan, Italy; 6 Center for Mind/Brain Sciences, University of Trento, Rovereto, Italy; 7 European Master’s in Clinical Linguistics (EMCL), University of Groningen, Groningen, The Netherlands; 8 EMCL University of Potsdam, Potsdam, Germany; 9 Neuroscience Institute, and Laboratory of Experimental Psychology, Faculty of Psychology, El Bosque University, Bogotá, Colombia; 10 Division of Neurosurgery, Structural and Functional Connectivity Lab Project, “S. Chiara” Hospital, APSS, Trento, Italy; 11 Department of Clinical Neuroscience, Institute of Neuroscience and Physiology, Sahlgrenska Academy, University of Gothenburg, Gothenburg, Sweden; 12 Department of Neuroscience, Neurology, Uppsala University, Uppsala, Sweden; 13 Cognition and Brain Plasticity Unit, Bellvitge Biomedical Research Institute (IDIBELL), University of Barcelona, Barcelona, Spain; 14 Department of Cognition, Development and Education Psychology, Barcelona, Spain; 15 Department of Neurology and Neurosurgery, Rudolf Magnus Brain Institute, University Medical Center of Utrecht, Utrecht, The Netherlands; 16 Department of Neurosurgery, Elisabeth-Tweesteden Hospital, Tilburg, The Netherlands; 17 Department of Neurosurgery, Addenbrooke’s Hospital, University of Cambridge, Cambridge, UK; 18 Department of Radiology, Addenbrooke’s Hospital, University of Cambridge, Cambridge, UK; 19 Neurosurgery Department, Hôpital Pasteur 2, University Hospital of Nice, Nice, France; 20 Radiology Department, Hôpital Pasteur 2, University Hospital of Nice, Nice, France; 21 Department of Neurosurgery, Sahlgrenska University Hospital, Gothenburg, Sweden; 22 Department of Clinical Neuroscience, Institute of Neuroscience and Physiology, Sahlgrenska Academy, Gothenburg, Sweden; 23 Department of Neurosurgery, Institution of Neuroscience, Uppsala University Hospital, Uppsala, Sweden; 24 Global Brain Health Institute, Trinity College Dublin, Dublin, Ireland; 25 Department of Cognitive Science, Johns Hopkins University, Baltimore, USA; 26 Department of Neurosurgery, Medical University Graz, Graz, Austria; 27 DACTIM, UMR CNRS 7348, Université de Poitiers et CHU de Poitiers, Poitiers, France; 28 Neuroradiology Department, University Hospital Santa Maria della Misericordia, Udine, Italy; 29 Department of Neurosurgery, CHU Toulouse, Toulouse, France; 30 ToNIC, Toulouse NeuroImaging Center, Université de Toulouse, Inserm, UPS, Toulouse, France; 31 Department of Neurosurgery, Medical Faculty, Heinrich Heine University, Düsseldorf, Germany; 32 VU University Medical Center, Amsterdam, The Netherlands; 33 Department of Neurosurgery, Sainte-Anne Hospital, Paris Descartes University, Sorbonne Paris Cité, Paris, France; 34 Inserm U894, IMA-Brain, Centre de Psychiatrie et Neurosciences, Paris, France; 35 Department of Neurosurgery, Hôpital Gui de Chauliac, Montpellier Medical University Center, Montpellier, France; 36 Institute of Neuroscience of Montpellier, INSERM U1051, University of Montpellier, Montpellier, France; 37 Department of Neurosurgery, Lariboisière Hospital, APHP, Paris, France; 38 University Paris 7, Paris, France; 39 IMNC, UMR 8165, Orsay, France; 40 Department of Neurosurgery, The General Infirmary at Leeds, Leeds, West Yorkshire, UK

**Keywords:** Low-grade glioma, Imaging in LGG, Minimal core of imaging, Response criteria

## Abstract

**Objective:**

Imaging studies in diffuse low-grade gliomas (DLGG) vary across centers. In order to establish a minimal core of imaging necessary for further investigations and clinical trials in the field of DLGG, we aimed to establish the status quo within specialized European centers.

**Methods:**

An online survey composed of 46 items was sent out to members of the European Low-Grade Glioma Network, the European Association of Neurosurgical Societies, the German Society of Neurosurgery and the Austrian Society of Neurosurgery.

**Results:**

A total of 128 fully completed surveys were received and analyzed. Most centers (n = 96, 75%) were academic and half of the centers (n = 64, 50%) adhered to a dedicated treatment program for DLGG. There were national differences regarding the sequences enclosed in MRI imaging and use of PET, however most included T1 (without and with contrast, 100%), T2 (100%) and TIRM or FLAIR (20, 98%). DWI is performed by 80% of centers and 61% of centers regularly performed PWI.

**Conclusion:**

A minimal core of imaging composed of T1 (w/wo contrast), T2, TIRM/FLAIR, PWI and DWI could be identified. All morphologic images should be obtained in a slice thickness of ≤ 3 mm. No common standard could be obtained regarding advanced MRI protocols and PET.

**Importance of the study:**

We believe that our study makes a significant contribution to the literature because we were able to determine similarities in numerous aspects of LGG imaging. Using the proposed “minimal core of imaging” in clinical routine will facilitate future cooperative studies.

**Electronic supplementary material:**

The online version of this article (10.1007/s11060-018-2916-3) contains supplementary material, which is available to authorized users.

## Introduction

Despite published standards and guidelines on treatment and follow-up of diffuse low-grade glioma (DLGG) patients, daily practice frequently demonstrates the inconsistency of imaging studies among centers [1, 2]. As the volume of DLGG publications increases, the usefulness of single-center studies will become more limited, as these may be difficult to replicate. It has been argued that evidence-based practice in the field of DLGG cannot be derived from the standard methodology of oncological randomized clinical trials [3, 4]. Considering the low prevalence of DLGG [5] and the long survival of patients [6], sufficient data might be better collected by networks of centers working together collaboratively. Numerous demographic parameters, oncomolecular features and imaging data (including imaging DLGG growth rates of follow-up MRIs) will be required. Rigorous evaluation of care is additionally needed, for example to prove that maximum safe resection is not only key to oncological outcome, but also to establish and maintain a best possible quality of life [7, 8]. The expectation that randomized oncological studies could add knowledge on these two questions is vanishing [3]. We are convinced that databases dedicated to DLGG research are required, which could include both retro- and prospective data.

The European Low-Grade Glioma Network (ELGGN) gathers surgical and neuroscience specialists from centers with dedicated teams treating DLGG patients. The network was founded 11 years ago to establish the link between all subspecialties involved in the field: neurosurgeons, neuro-oncologists, radiation therapists, neuropathologists, oncomolecular biologists, neuroradiologists, anaesthesiologists, speech therapists, neuropsychologists, and neuroscientists involved in functional brain mapping. Several collaborative studies have been previously published [3, 9–11].

The ELGGN is a powerful platform to address major issues in the management of DLGG. A survey [3] has been created in preparation of the 2015 Annual Meeting, which met the goal to identify points of consensus in patient management. Thus, the network should allow to highlight relevant questions for future studies and establish landmark projects in the interdisciplinary treatment of DLGG.

Following the initial survey, we now aimed to establish a comprehensive imaging survey, in order to investigate the consistency of DLGG imaging in specialized centers across Europe and to identify a “minimal core of imaging” to facilitate cooperative imaging projects within the network.

## Methods

An online survey was created by a group of experts in imaging and treatment of DLGG. The use of published imaging guidelines [1, 12, 13] was emphasized and local availability and usage of advanced imaging modalities was added to the survey. The survey was formatted on Survey Grid (EvaSys, Electric Paper Evaluationssysteme GmbH Lüneburg, Germany) and sent to all members of the ELGGN, the European Association of Neurosurgical Societies (EANS) plus the German (DGNC) and Austrian Society of Neurosurgery (ÖGNC). All recipients were members of the respective societies and it was specified that the questionnaire should be filled out by a multidisciplinary team. Participants were not asked to detail how any disagreements were adjudicated, precluding analysis of response heterogeneity at the center level. The survey contained 45 items (see Supplement 1), divided in descriptive data (i.e. amount of DLGG treated per year), radiological details (i.e. MRI sequences that are routinely performed) and questions regarding advanced imaging techniques (i.e. diffusion weighted imaging (DWI)).

In order to distinguish between dynamic susceptibility contrast perfusion imaging (DSC, named PWI in our survey) and dynamic contrast-enhanced MR perfusion (DCE, named Perfusion in our survey), two perfusion modalities were included in the survey, however, the question was answered inappropriately, showing that the nomenclature used for this particular question was misleading. Technical data on MRI scanner manufacturers, acquisition and recovery times, magnetic field strength and contrast agent dosage were not acquired. Further, the survey did not evaluate scientific justification of imaging protocols, it depicted a common denominator across many centers.

Further sections of the survey inquired the routine use of non-invasive mapping (i.e. resting state functional MRI (rs-fMRI) or navigated transcranial magnetic stimulation (nTMS)) and follow-up imaging protocols. The survey consisted of 28 single-choice, 7 multiple-choice and 10 items for free text answers.

In total, 148 data sets were received for detailed analysis. Data from outside Europe’s geographical extension (n = 14) and incomplete surveys (n = 6) were excluded. Overall, 128 fully completed surveys were analyzed descriptively for this study. For additional information and geographical details of the respondents see Table 1.

**Table 1:** Distribution of centers per country and average number of treated DLGG

	Number of centers responded	%	Avg. no of LGG per center and year	Range
Country of practice				
Germany	40	31	20	5–50
Italy	14	11	10	5–90
France	9	6	25	3–100
Switzerland	8	7	20	5–100
United Kingdom	8	6	30	10–60
Austria	7	5	20	6–40
Spain	7	5	5	3–20
Netherlands	6	5	25	15–40
Portugal	4	3	20	2–20
Belgium	3	2	15	15–30
Greece	3	2	25	10–30
Poland	3	2	25	20–30
Czech Republic	2	2	15	15
Russian Federation	2	2	55	50–60
Serbia	2	2	15	10–20
Sweden	2	2	20	20
Bulgaria	1	< 1	10	10
Denmark	1	< 1	20	20
Hungary	1	< 1	n/a	n/a
Lithuania	1	< 1	30	30
Norway	1	< 1	30	30
Romania	1	< 1	10	10
Turkey	1	< 1	20	20
Ukraine	1	< 1	5	5
Use of 3T imaging	128			
Always	29	22.8		
If available	59	46.5		
Only 1.5T	39	30.7		
Identical MR scanner				
Yes	25	20.0		
No	24	19.2		
Mostly yes	72	57.6		
Mostly no	4	3.2		

			Total number of patients	
Slice thickness of T1 imaging (mm)				
< 1.5	66	53.7	**–**	1585
1.6–3	44	35.8	**–**	781
> 3	13	10.6	**–**	356
Slice thickness of T2 imaging (mm)				
< 1.5	40	32.8	**–**	1027
1.6–3	54	44.3	**–**	1133
> 3	28	23.0	**–**	542
Imaging intervals in relation to the amount of residual disease				
No remnant (weeks)				
< 12	3	2.4		
12–24	87	68.5		
> 24	15	11.8		
52	4	3.1		
< 10 ml remnant (weeks)				
< 12	4	3.1		
12–24	91	71.7		
> 24	12	9.4		
52	0	0.0		
11–15 ml remnant (weeks)				
< 12	4	3.1		
12–24	95	74.8		
> 24	9	7.1		
52	0	0.0		
> 15 ml remnant (weeks)				
< 12	7	5.5		
12–24	97	76.4		
> 24	4	3.1		
52	0	0.0		
Unresectable LGG (weeks)				
< 12	1	0.8		
12–24	97	76.4		
> 24	6	4.7		
52	3	2.4		

Use of imaging infrastructure and average slice thickness. Imaging intervals with respect to the amount of residual disease

## Results

### Basic information

**Fig. 1:**
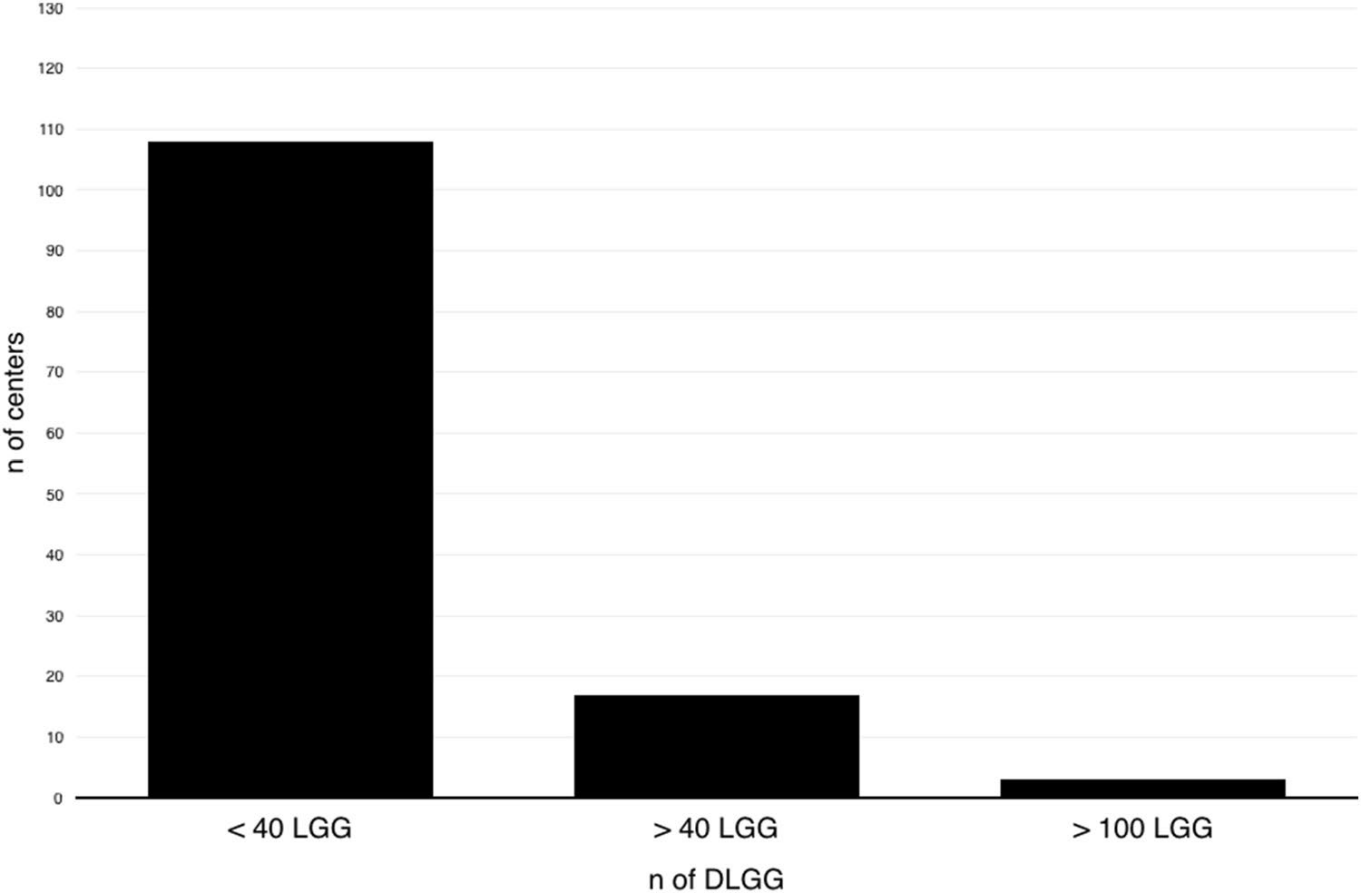
Distribution of annual DLGG throughout participating centers. Most centers treat less than 40 DLGG per year

The majority of responding teams worked in an academic hospital (n = 96, 75%), 19% (n = 25) were based in community hospitals, and 6% (n = 8) were located in a private hospital environment. Half of the centers (64, 50%) adhered to a dedicated MRI protocol for DLGG, while the others (64, 50%) did not. There was a broad range in the reported operative activity for each center (2–100 cases/year, average 25). The majority of centers treated less than 40 DLGG per year (n = 108, 85%), whereas only 17 centers (13%) reported to treat more than 40. Three centers (2%) can be considered very high volume centers with 100 DLGG per year (see Fig. 1). A total of 3032 DLGG are managed annually by the responding centers, the majority (1714, 62%) of which are treated in centers with dedicated DLGG programs compared to 1068 (38%) in centers without. Of the centers treating 40 or more DLGG per year 65% used a dedicated DLGG protocol.

### Physicians involved in treatment of DLGG

The survey showed that there is a variation in the composition of the multidisciplinary teams involved in DLGG management. The majority of centers (84%) discussed their patients either before (59, 47%) or after surgical treatment (47, 37%). Interestingly, 20 centers (16%) refrained from presenting every surgically treated DLGG in a multi-disciplinary tumor board, and 16 of these only discussed them if adjuvant treatment was advocated. Multi-disciplinary tumor boards consisted of several specialties: neurosurgeons were present in 99%, followed by neuroradiologists (90%), and radiation oncologists (87%). Medical oncologists participated in 80% of neuro-oncological tumor boards, an additional 64% included a specialized neuro-oncologist. Nuclear medicine specialists, however, were available at the tumor board in only 32% of centers.

### Imaging

More than half (52%) of the centers routinely used any recent MR imaging for treatment decisions and surgical treatment, without performing an MRI according to their own dedicated protocol. The imaging had to be carried out in a specialized neuroradiology unit (i.e. in a university hospital) in 17% of centers and 31% of centers always scanned their own dedicated protocol. The particular sequences applied by the center are summarized in Table 2. T1 imaging without and with Gadolinium contrast (T1 wo/w) and T2 weighted imaging was obtained in every center (100%), whereas TIRM (turbo inversion resonance magnitude) and FLAIR (fluid attenuation inversion recovery) are performed in 20 and 98%, respectively. Centers used a slice thickness < 1.5 mm in 52% of T1 and 32% of T2 images and a slice thickness of ≤ 3 mm in 89% of T1 and 78% of T2 images. Most of the centers (89%) obtained DWI in every patient with an additional 80% obtaining apparent diffusion coefficient (ADC) maps routinely. Almost two-thirds of the respondents (61%) applied perfusion weighted imaging (PWI) in daily routine.

**Table 2:**
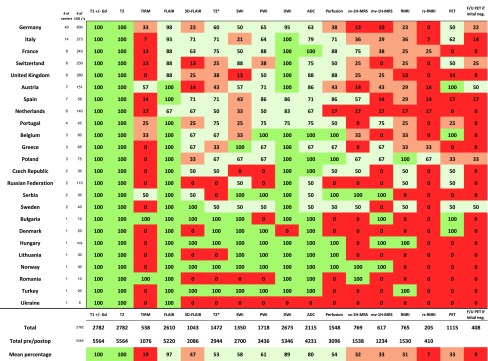
Availability of MR-sequences in %

*T1+/−Gd* T1 weighted imaging with and without Gadolinium contrast, *TIRM* turbo inversion resonance magnitude, *FLAIR* fluid attenuated inversion recovery, *3D-FLAIR* multiplanar reconstruction of FLAIR, T2* gradient-echo T2 with susceptibility, *SWI* susceptibility weighted imaging, *PWI* perfusion weighted imaging, *DWI* diffusion weighted imaging, *ADC* automated diffusion coefficient, *sv-1H-MRS* single-voxel Proton magnetic resonance spectroscopy, *mv-1H-MRS* multi-voxel Proton magnetic resonance spectroscopy, *fMRI* functional MRI, *rs-fMRI* resting-state functional MRI, *PET* positron emission tomography

Follow-up imaging of DLGG always included volumetric analysis (segmentation and approximation) in 45 centers (35%) and linear measurement (3 axes on MRI) in 58 centers (45%), and 26 centers (20%) evaluated the deformation of present lesions or changes in shape to identify progression or regression. All measurements were performed by neuroradiologists in 59% opposed to 24% by neurosurgeons and 3% by neurooncologists. In 14% of centers, all members of the team performed measurements.

For interpretation of the response in DLGG, the RANO criteria [13] were used “always” in 15% of centers and “most of the time” in 46% of centers. 17% used the published criteria “hardly ever” and 22% refused to utilize them.

Although 81% of centers specified to adjust their imaging intervals according to the tumor’s previous growth rate, more than 75% of centers perform follow-up imaging in intervals of 12–24 weeks (see Table 1) in all cases presented, regardless of the amount of residual tumor.

### Advanced imaging

Additional advanced sequences, like MR spectroscopy (MRS) were handled variably throughout the centers. One-third of centers routinely obtained data from single-voxel spectroscopy, another third applied a multi-voxel spectroscopy in their MRI protocol.

The question “Do you perform amino acid PET in suspected low-grade glioma?” was answered positively in 33% of all respondents. However, centers tend to discard PET imaging in case of initially negative PET scans. Under these circumstances only 9% would repeat PET imaging later. Interestingly, some countries like Austria and Belgium had a 100% rate of initial PET imaging, whereas French centers performed no PET. Assessment of treatment response and progression was mostly (80%) done with MRI (T2/FLAIR for response, T1wo/w for progression), whereas 14% relied on the combination of MRI and amino-acid PET (aaPET; if initially positive). Six percent of centers performed MRI and aaPET regardless of initial PET presentation. The survey investigated which imaging the centers would rely on to decide whether the tumor has undergone anaplastic transformation. Unsurprisingly, there is no consensus on the imaging modalities used for detection of anaplastic transformation. Most centers used combinations of either T1wo/w with MRS and PWI or T1wo/w with FET-PET (see Table 3).

**Table 3:** Choice of imaging modalities for detection of malignant transformation

	n	%
T1 + PWI + MRS	23	18
T1 + FET-PET	20	16
T1 + PWI	20	16
T1	19	15
T1 + PWI + FET-PET	10	8
T1 + PWI + MRS + FET-PET	8	6
T1 + MRS	4	3
T1 + MRS + FET-PET	3	2
T1 + PWI + MRS + other	3	2
FET-PET	3	2
PWI + MRS	3	2
T1 + PWI + other	2	2
T1 + other	2	2
other	8	6
Other		
Biopsy/resection	7	6
Evaluate changes in growth rate/volumetric expansion	3	2
F-DOPA PET	1	1
Arterial-spin labelling MRI	1	1
DWI/ADC	1	1

The number of MRI studies that would be available for further investigation was calculated for every MR sequence based on the number of patients treated annually in all responding centers. Up to 2800 studies should be available for analysis every year (see Table 2).

### Functional MRI and non-invasive brain mapping

The survey demonstrated a wide heterogeneity regarding the use of functional MRI (fMRI) for patients with DLGG.

Thirty-one percent routinely used fMRI for every patient, while resting-state fMRI is acquired in only 7%. Half of the centers (50%) used fMRI for both, clinical and research purposes, 42% exclusively clinical, and 6% only for research. A minority of 2% used fMRI for didactic purposes in training of students or residents.

The final part of the survey evaluated brain mapping and the respective technique of choice: fMRI, nTMS, or intraoperative direct electrical cortical and subcortical stimulation. 102 centers (80%) preferred invasive intraoperative direct electrical cortical and subcortical stimulation over noninvasive procedures. 8 centers (6%), however, would have chosen nTMS, whereas 18 centers (14%) preferred fMRI. The number of centers using magnetoencephalography (MEG) was low, which fits the distribution of the technique. 87% of centers don’t use MEG or do not own one, although 71% recognized the scientific possibilities of MEG or thought it would be nice to have.

## Discussion

Our survey revealed a high level of homogeneity in DLGG imaging workup throughout Europe. Nonetheless, we were able to identify heterogeneities that need to be highlighted. Of note, questions were not designed as detailed individual cases and we acknowledge that this method might have provoked heterogeneity of responses.

### Minimal core of imaging

Ellingson et al. [1] proposed a standardized brain imaging protocol for tumor patients. We support the principle of creating standardized protocols for DLGG patients. This should include minimum imaging datasets, recommended imaging frequency and recommendations about additional sequences. The goal of establishing a minimal core of imaging would be to allow further investigations with uniform imaging throughout different countries. This would enable to include numerous patients in future prospective cohorts, ensuring a sufficient volume of data to perform big data analysis [14].

The minimal core of imaging in DLGG needs to take regional and national differences regarding technical standards and reimbursement into account. Therefore, the following imaging algorithm is proposed: MR imaging should incorporate sequences for morphologic descriptive analysis and those focusing on potential malignant transformation. T1wo/w and T2 images were used by all centers and therefore represent the cornerstone of morphologic imaging. In addition, TIRM or FLAIR sequences should be obtained and can also be used for volumetric assessment of DLGG. Although several recommendations set the minimum level of slice thickness in T2 and TIRM/FLAIR imaging to ≤ 4 mm [1], it is known that slice thickness is key for accurate volumetric and treatment response assessment [15–17]. Perfusion weighted imaging (PWI) is used frequently for assessment of treatment response and detection of anaplastic transformation [18, 19]. In our survey, response and transformation were predominantly determined with MRI. T1 (with contrast) and PWI were used in 87 and 55%, respectively. Diffusion weighted imaging (DWI), and especially the computed ADC has been used to investigate malignant transformation [20] and is applied in up to 90% of centers.

We therefore recommend that the minimal MRI sequence dataset should consist of T1wo/w, T2 and TIRM/FLAIR, all in low slice thickness (≤ 3 mm) to facilitate volumetric assessment and further include PWI and DWI to predict malignant transformation. Based on this survey, we cannot make any recommendation regarding the use of additional advanced imaging techniques (spectroscopy, fMRI, PET).

### Imaging intervals

More than 75% adhered to follow-up imaging intervals of 12 to 24 weeks and 81% adjusted their imaging intervals depending on the initial growth rate of the tumor [21], which is in accordance with current guidelines [2]. It has been shown that the velocity of tumor expansion is a strong predictor of the patient’s prognosis [21]. However, no studies have addressed the question of how often MRI should be obtained during follow-up, especially with regard to the heterogeneity of DLGG. Although early postoperative FLAIR is known to overestimate the volume of residual tumor [22], most authors recommend an early postoperative MRI within 72 h [23–25] to determine the extent of resection and visualize possible postoperative complications. Follow-up imaging intervals of 12–24 weeks are recommended with longer intervals for cases of “less aggressive” tumours [1, 13, 26]. The definition of “aggressiveness”, however, varies in the literature.

### Standardized response assessment protocols

Only 15% of the centers reported to use RANO criteria for low-grade gliomas [13] thoroughly in all their DLGG cases for the interpretation of treatment response, while 46% of centers do so “most of the time”. Thus, 39% of centers do not routinely apply these criteria. RANO criteria include T1wo/w, T2/FLAIR, development of new lesions, clinical status, and steroid use in order to categorize treatment effects in complete response, partial response, stable disease, and progressive disease. Since DLGG constitutes a slowly progressive disease, these criteria were defined in order to achieve a standardized common ground for the definitions of treatment responses and endpoints in clinical trials. Measurement of tumor diameter has various shortcomings, not only the high intra- and interobserver variability [27], but also the known problem of head positioning during acquisition of the MRI [16, 17]. Then, assuming that many centers are performing volumetric assessment of tumors, how should treatment response be defined? Translating volume to diameter (D = (2 × V)^(1/3)) is a fundamental step. In contrast to the assessment of tumor diameters, the concept of volume-derived mean diameter overcomes the above mentioned confounding factors. Moreover, the curve showing evolution of mean diameter with time can be easily analyzed by applying a linear fit. Following the curve of the diameter as a function of time is a more sensitive way to monitor treatment response than applying RANO criteria [21, 28, 29]. Indeed, the major concern about RANO criteria is that pretreatment dynamics is not integrated in the definition of the different response categories. However, it seems obvious that putting down the tumor growth rate to 0 mm/year with chemotherapy, while its pretreatment value was 6 mm/year, should be interpreted as a response, whereas RANO criteria would interpret this as “stable disease”.

In contrast to this highly standardized protocol, our survey focused more on the clinical routine in an attempt to accurately reflect every day practice in the treatment of DLGG across Europe. Although the highly standardized approach provided by the RANO criteria is not systematically applied, our data demonstrate that at least the imaging studies, comply in most centers with the RANO-defined protocols.

Additionally, other factors should also be interpreted into our decision making, such as cognitive testing, psychological burden of disease, and seizure activity.

### Malignant transformation

There is no consensus regarding the radiological malignant transformation in DLGG throughout Europe. Newly apparent contrast enhancement indicating breakdown of the blood brain barrier represents the classical sign of malignant transformation (MT) of these tumors [30], but preceding changes in advanced MRI investigations may allow identification of patients at risk up to 12 months earlier [31]. Simple measurement of growth rate [19, 32, 33] and integration of MR spetroscopy [18, 19, 34] are used routinely in most of the centers as predictors of tumor transformation. Both, proton- and phosphorus spectroscopy, available in numerous centers, have been proven to correlate with Ki67 and IDH1 mutation [35]. Perfusion measurements, and in particular the determination of relative cerebral blood volume (rCBV), seems to correlate with the vascularity determined at histopathological examination [31]. Arising of lactate resonance is predictive of the increase of rCBV up to 1.75, this parameter is predictive for a dramatic decrease of overall survival [36–38], and are indicators of MT that may be identified months before apparent contrast enhancement. Likewise, ADC can be used for discrimination of tumor subtypes and raising suspicion of malignization [20]. Nevertheless, both techniques are only supported by a low level of evidence [20].

### Positron emission tomography (PET)

The use of amino acid PET (aaPET) was surprisingly variable between centers and countries. While Austria and Belgium performed PET in 100% of cases, none of the Dutch or French centers used PET at all. In contrast to patients with WHO grades III/IV gliomas, the evidence for aaPET to monitor patients with WHO grade II gliomas is limited [39, 40]. Most WHO grade II gliomas are nonenhancing with infiltrating tumor borders, and so several studies demonstrated the usefulness of aaPET in defining tumor extent or malignant transformation [41–45]. This has been demonstrated and validated in series for 11C-MET, 18F-FET, and 18F-FDOPA PET [46]. Although MRI is the standard of care in following DLGG patients, its reliability in distinguishing tumor tissue from treatment effects is limited [47]. Transient blood–brain barrier alteration with contrast enhancement after radiotherapy with or without concomitant Temozolomide, for example, can mimic tumor progression. There are numerous reasons for the restricted application of PET: (1) While 18F-FDG is used routinely, access to aaPET is limited [46]. (2) Another obstacle to withhold patients and healthcare professionals from PET is limited reimbursement. In our survey, 9% of centers repeated aaPET scans during follow-up, even if they were initially negative. However, there are several groups advocating to perform aaPET in all cases of DLGG [48], notwithstanding the initial uptake behavior.

### fMRI and noninvasive neurophysiological imaging

fMRI has repeatedly been shown to harbor a low specificity and sensitivity for any presurgical evaluation [49–53]. Since the tumor itself impairs oxygenation levels in its surrounding fMRI is not a reliable surrogate marker for neuronal activity in DLGG patients [50, 51, 54]. Nevertheless, eloquence is thoroughly based on fMRI in as much as 16% of centers.

Yet, 80% of European centers prefer invasive intraoperative direct electrical cortical and subcortical stimulation over noninvasive procedures for the determination of eloquent cortex, which reflects commonly agreed practice and level of evidence [55–57].

With the still small but increasing distribution of nTMS, some centers chose this technique for surgical decision making, which also illustrates the highly specialized nature of the enrolled centers. Although nTMS is a noninvasive modality, several reports proved not only the accuracy but also the feasibility of using it as a highly reliable tool for presurgical planning and intraoperative navigation [23, 58, 59] of primary motor functions. While non-invasive mapping can be reliably performed for primary motor functions (task-based fMRI, nTMS), this is currently not true for higher order functions (movement coordination, language, spatial consciousness, mentalizing, …) [60].

MEG was also rarely used. Nonetheless, despite its limited availability and high costs, its usefulness for presurgical planning and follow-up has repeatedly been reported [61–63].

Irrespective of the used modality, noninvasive evaluation of eloquent function, especially if adjacent to or within the tumor is mandatory in order to identify the optimal time point for re-resection. This is even more relevant in the contrast of tumor-induced cortical reorganization potentially allowing gross total resection of previously unresectable tumors [64–68].

In the near future, ongoing developments might be capable to achieve a change in imaging practice. Alongside with huge developments of machine learning using conventional MRI, which suppose the robustness of standardized sequences; an increased interest of metabolic multinuclear MR imaging and integration of multiparametric data into realistic metabolic-dynamic mathematical models is noticed.

## Strengths and limitations

The survey solely focused on imaging modalities for preoperative workup and follow up investigations of DLGG patients. The large number of centers involved and the high conformity of surgical treatment within participants of the ELGGN represent the major strength of this study. Up to 2800 MRIs per year would be available in this network. Online surveys are limited by the accuracy of the given statements and their representation of a multidisciplinary team cannot be guaranteed in online survey, which limits the reliability of the results. Questions were not designed as detailed individual cases and we acknowledge that this method might have provoked heterogeneity of responses. Additionally, the study did not aim to investigate the outcome of DLGG treatment.

## Conclusion

It appears mandatory to standardize the initial management and follow-up of DLGG in order to maximize the number of included patients in future multicentric studies. If a certain proximity of imaging protocol throughout Europe is already present, this work emphasizes the need to clarify important questions such as assessment of treatment response or detection of DLGG malignant transformation. ELGGN can help to resolve important issues and to promote a better care for patients suffering from DLGG.

## Electronic supplementary material

Below is the link to the electronic supplementary material.

Supplementary material 1 (PDF 168 KB)
