# Supplementary material for: Imaging practice in low-grade gliomas among European specialized centers and proposal for a minimum core of imaging
Source: J Neurooncol. 2018 Jul 10;139(3):699–711. doi: 10.1007/s11060-018-2916-3 (PMC6132968; doi:10.1007/s11060-018-2916-3)
Supplement: Supplementary file 1 — Supplementary material 1 (PDF 168 KB) [file 11060_2018_2916_MOESM1_ESM.pdf]

Questionnaire European Low Grade Glioma Network

Type of Hospital:

- ☐ academic
- ☐ communal
- ☐ private (including private practice)

What is the total amount of diffuse low-grade gliomas (dLGG) in your practice per year?

Country of practice:

Do you have a dedicated „LGG-program“ in your practice?

- ☐ Yes
- ☐ No

When do you discuss a case in a dedicated interdisciplinary board (tumor-board)?

- ☐ Never
- ☐ Before every surgery
- ☐ After every surgery
- ☐ If adjuvant treatment is advocated

Which specialities are present in your interdisciplinary (tumor-) board?

- ☐ Neurosurgery
- ☐ Neurology (Neurooncologists)
- ☐ Medical Oncology
- ☐ Radiation Oncology
- ☐ Nuclear Medicine
- ☐ Neuroradiology
- ☐ Neuropathology

In your routine practice, do you use external imaging?

- ☐ No, we always scan our own protocol.
- ☐ Yes, if the imaging was done in a neuroradiology center (University Hospital)
- ☐ Yes, we use any recent MRI

What is contained in your standard imaging protocol?

- ☐ T1 with / without Gd-contrast
- ☐ T2
- ☐ TIRM
- ☐ FLAIR
- ☐ 3D-Flair
- ☐ T2*
- ☐ Susceptibility weighted Imaging (SWI)
- ☐ Perfusion weighted Imaging (PWI)
- ☐ Diffusion weighted Imaging (DWI)
- ☐ ADC
- ☐ Perfusion
- ☐ single voxel 1H-spectroscopy
- ☐ multivoxel 1H-spectroscopy
- ☐ fMRT
- ☐ resting stare fMRT

Do you always use the identical MRI scanner for one patient during follow-up investigations?

- ☐ Yes
- ☐ No
- ☐ Mostly yes
- ☐ Mostly no

Do you always use a 3T scanner?

- ☐ Yes, always
- ☐ If available, we use a 3T scanner
- ☐ No, 1,5 T is acceptable

What is the standard imaging slice thickness for T1 imaging in LGG?

- ☐ <1.5 mm
- ☐ 1.6 - 3 mm
- ☐ 3 mm

What is the standard imaging slice thickness for T2 imaging in LGG?

- ☐ <1.5 mm
- ☐ 1.6 - 3 mm
- ☐ 3 mm

Do you obtain 1H spectroscopy of both hemispheres?

- ☐ Yes
- ☐ No

Do you perform amino acid PET in suspected low-grade glioma?

- ☐ Yes
- ☐ No

If the initial amino acid PET was negative, do you perform follow-up amino acid PET imaging?

- ☐ Yes
- ☐ No

For assessment of treatment response and progression you use?

- ☐ MRI only
- ☐ amino acid PET only (if initial PET was positive)
- ☐ always MRI + aaPET (if initial PET was positive)
- ☐ always MRI + aaPET (regardless of initial PET)

Questionnaire European Low Grade Glioma Network [Fortsetzung]

How do you detect anaplastic transformation?

- ☐ T1 w/o contrast
- ☐ Perfusion weighted imaging
- ☐ 1H spectroscopy
- ☐ FET-PET
- ☐ other

If other, please specify

How do you rate the following combination of characteristics in your department: (1) a decrease of the area of non-enhancing lesion on T2 or FLAIR imaging between 25% - 50% compared with baseline; (2) no new lesions, no new T2 or FLAIR abnormalities apart from those consistent with radiation effect, and no new or increase enhancement; and (3) patients should be on a corticosteroid dose that should not be greater than the dose at time of baseline scan, and should be stable or improved clinically.

- ☐ Complete Response
- ☐ Partial Response
- ☐ Minor Response
- ☐ Stable Disease
- ☐ Progression

Do you analyze DTI for 3D-fiber tracking in every patient?

- ☐ Yes
- ☐ No

What software do you use for analysis of DTI?

Which mathematical model do you use for analysis?

- ☐ Unknown
- ☐ Probabilistic approach (including the uncertainty of the estimation, which results in probability maps representing the likelihood of a voxel being part of a fiber and provides the multiple possible fiber directions emanating from each seed)
- ☐ Deterministic approach (Deterministic tractography aims to model the data and, in practical terms, can be thought of as generating/reconstructing one fiber from each seed)
- ☐ Atlas based

If you do fMRI, is it only for clinical answers or research?

- ☐ clinical only
- ☐ research only
- ☐ both clincal and research
- ☐ for didactic purpose

Which fMRI paradigms do you use routinely?

- ☐ motor paradigm (i.e. finger tapping)
- ☐ somatosensory stimulation
- ☐ speech - word generation / naming task
- ☐ speech - synonyms / odd one out
- ☐ speech - semantic comparison
- ☐ other

If other, please specify

Do you routinely analyze resting state fMRI?

- ☐ Yes
- ☐ No

If you would have to choose between fMRI, navigated transcranial stimulation (nTMS) and direct cortical stimulation (DCS), what would you prefer?

- ☐ fMRT
- ☐ nTMS
- ☐ DCS

If you do navigated transcranial magnet stimulation (nTMS), is it only for clinical answers or research?

- ☐ clinical only
- ☐ research only
- ☐ both clinical and research

How does your decision on nTMS influence your daily practice?

- ☐ more awake surgery
- ☐ more intraoperative monitoring
- ☐ more resective surgery
- ☐ less resective surgery or biopsy
- ☐ less awake surgery
- ☐ less intraoperative monitoring
- ☐ doesn’t influence at all

Do you use the exact RANO criteria (van den Bent, Wefel et al. 2011) for LGG?

- ☐ always
- ☐ mostly
- ☐ almost never
- ☐ never

Do you always assess follow-up imaging by:

- ☐ volumetric analysis (segmentation)
- ☐ volumetric analysis (approximation)
- ☐ linear measurement in 3 axes of the MRI
- ☐ deformation of the lesions / changes in shape

Questionnaire European Low Grade Glioma Network [Fortsetzung]

Who does the measurements?
- ☐ Neurosurgeon
- ☐ Neurooncologist
- ☐ Neuroradiologist
- ☐ All members of the team

Are measurements always done by the same investigator?
- ☐ Yes
- ☐ No

What is your imaging interval in weeks in cases of diffuse low-grade glioma (without suspected transformation) with:

no remnant

[ ]

10ml tumor remnant

[ ]

11 - 15ml tumor remnant

[ ]

> 15ml remnant

[ ]

unresectable LGG

[ ]

Does this interval vary according to the growth rate?
- ☐ Yes
- ☐ No

Do you perform longitudinal fMRI study (e.g. before and after surgery) to investigate neuroplasticity?
- ☐ Yes
- ☐ No

Do you combine different methodologies to increase the reliability of functional imaging - as for example fMRI or TMS and DTI?
- ☐ Yes
- ☐ No

Do you use a databank of all MRIs in DICOM format?
- ☐ Yes, on hospital PACS
- ☐ Yes, on Harddrive
- ☐ No

Do you use magnetoencephalography (MEG) in your center?
- ☐ for language mapping
- ☐ for language lateralization
- ☐ for motor mapping
- ☐ we have one but do not use it
- ☐ we don’t have a MEG

What is your opinion on MEG in general:
- ☐ we currently try to get one
- ☐ I don’t see any value for neuro-oncology
- ☐ It is a crucial technique for clinical decision making
- ☐ It is nice to have but you don’t really need it
- ☐ It is clinically not relevant but scientifically interesting, even for neuro-oncology

Beyond clinical considerations, do you work with a research team involved in neuroimaging?
- ☐ Yes, Biostatisticians
- ☐ Yes, Bioinformaticians
- ☐ Yes, Physicists
- ☐ Yes, Mathematicians
- ☐ No

Bibliography

van den Bent, M. J., J. S. Wefel, D. Schiff, M. J. B. Taphoorn, K. Jaeckle, L. Junck, T. Armstrong, A. Choucair, A. D. Waldman, T. Gorlia, M. Chamberlain, B. G. Baumert, M. A.
Vogelbaum, D. R. Macdonald, D. A. Reardon, P. Y. Wen, S. M. Chang and A. H. Jacobs (2011). "Response assessment in neuro-oncology (a report of the RANO group): assessment of outcome in trials of diffuse low-grade gliomas." The Lancet Oncology 12(6): 583-593.
